# Root and shoot variation in relation to potential intermittent drought adaptation of Mesoamerican wild common bean (*Phaseolus vulgaris* L.)

**DOI:** 10.1093/aob/mcy221

**Published:** 2018-12-31

**Authors:** Jorge C Berny Mier y Teran, Enéas R Konzen, Viviana Medina, Antonia Palkovic, Andrea Ariani, Siu M Tsai, Matthew E Gilbert, P Gepts

**Affiliations:** 1 University of California, Department of Plant Sciences/Mail Stop 1, Section of Crop & Ecosystem Sciences, Davis, CA, USA; 2 Centro de Energia Nuclear na Agricultura (CENA), Universidade de São Paulo, Piracicaba, SP, Brasil

**Keywords:** Crop wild relative, domestication, ecological genomics, genome-wide association, genotyping by sequencing, georeferencin, local climate adaptation, plant growth, single-nucleotide polymorphism

## Abstract

**Background:**

Wild crop relatives have been potentially subjected to stresses on an evolutionary time scale prior to domestication. Among these stresses, drought is one of the main factors limiting crop productivity and its impact is likely to increase under current scenarios of global climate change. We sought to determine to what extent wild common bean (*Phaseolus vulgaris*) exhibited adaptation to drought stress, whether this potential adaptation is dependent on the climatic conditions of the location of origin of individual populations, and to what extent domesticated common bean reflects potential drought adaptation.

**Methods:**

An extensive and diverse set of wild beans from across Mesoamerica, along with a set of reference Mesoamerican domesticated cultivars, were evaluated for root and shoot traits related to drought adaptation. A water deficit experiment was conducted by growing each genotype in a long transparent tube in greenhouse conditions so that root growth, in addition to shoot growth, could be monitored.

**Results:**

Phenotypic and landscape genomic analyses, based on single-nucleotide polymorphisms, suggested that beans originating from central and north-west Mexico and Oaxaca, in the driest parts of their distribution, produced more biomass and were deeper-rooted. Nevertheless, deeper rooting was correlated with less root biomass production relative to total biomass. Compared with wild types, domesticated types showed a stronger reduction and delay in growth and development in response to drought stress. Specific genomic regions were associated with root depth, biomass productivity and drought response, some of which showed signals of selection and were previously related to productivity and drought tolerance.

**Conclusions:**

The drought tolerance of wild beans consists in its stronger ability, compared with domesticated types, to continue growth in spite of water-limited conditions. This study is the first to relate bean response to drought to environment of origin for a diverse selection of wild beans. It provides information that needs to be corroborated in crosses between wild and domesticated beans to make it applicable to breeding programmes.

## INTRODUCTION

Most plant breeding relies on the genetic diversity of the domesticated gene pool to assemble and recombine the different traits that make up improved, elite varieties, mainly through selective cross-hybridization. Yet in many (but not all) crops domesticated diversity only represents a fraction of what is available in the wild ancestral gene pool, due to a combination of evolutionary processes including genetic bottlenecks and drift, selection and migration (Gepts, 2004, 2014). Hence, there has been a growing interest in using wild relatives for the purpose of developing improved crop cultivars and ‘re-domesticating’ crops to broaden the genetic diversity included in the domesticated gene pool. Crop wild relatives, especially the ancestral forms, have been subjected to biotic and abiotic stresses on an evolutionary time scale prior to domestication. Consequently, these wild types are likely adapted to the stresses they have been confronted with. This raises the following question: to what extent do adaptations of the domesticated types reflect adaptations of their wild ancestor or were these adaptations acquired post-domestication through *de novo* variation or local gene flow with wild relatives?

In this report, we focus on the common bean (*Phaseolus vulgaris*), the main grain legume for direct human consumption (Gepts *et al.*, 2008). More than half of the area of common bean production is grown under drought conditions and, after diseases, drought stress is the second most important factor that reduces productivity (Singh, 2001; Beebe *et al.*, 2013a). In bean, drought stress not only causes significant reduction in biomass, seed weight and yield, but also changes the nutritional quality of the seeds (Kigel, 1999; Rosales-Serna *et al.*, 2004; Beebe *et al.*, 2013b). Breeding for higher yields under drought would increase the area suitable for bean production by 31 % above the current distribution (Beebe *et al.*, 2011).

**Fig. 1.**
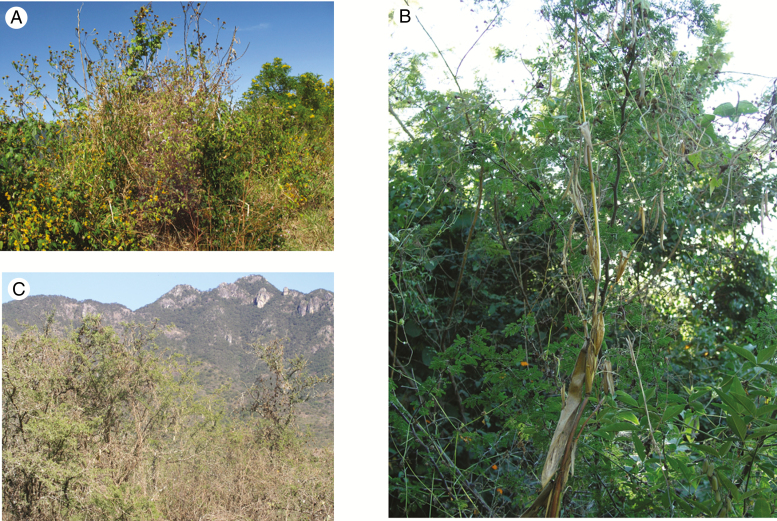
Habitat of Mesoamerican common bean. (A) Costa Rica. (B) Colima, Mexico, growing alongside teosinte. (C) Jalisco, Mexico. Photo credits: (A) Daniel Debouck; (B, C) Paul Gepts.

The genus *Phaseolus* originated in Mesoamerica and gave rise to five domesticated species, the most important of which is common bean (Ariani *et al.*, 2017; Bitocchi *et al.*, 2017). Common bean was domesticated twice from a diverged ancestral wild type in Mesoamerica and the southern Andes (Kwak and Gepts, 2009; Ariani *et al.*, 2017). Wild common bean (Fig. 1) grows on hillsides and high slopes, in rich, well-drained soils, over shrubs and trees as physical support, in natural or human-disturbed areas from 500 to 1900 m above sea level and in a range of rainfall from 500 to 1850 mm year^−1^ (Fig. 1) (Gentry, 1969; Freytag and Debouck, 2002). Because of adaptation to a wide range of precipitation and year-to-year variation of rainfall, we h)ypothesized that wild beans possess a range of mechanisms for drought adaptation.

Wild common bean populations from the Mesoamerican gene pool represent a suitable model to study the ecological genetics related to drought adaptation because they grow in a wide range of climatic and soil conditions and topographies. There is also a representative, georeferenced germplasm resulting from multiple explorations over several decades starting in the 1940s. The availability of whole-genome reference sequences (Andean: Schmutz *et al.*, 2014; Mesoamerican: Vlasova *et al.*, 2016) and geographical information systems have allowed the quantification of relationships between genetic variation and climatic or geographical variation, allowing greater understanding of local adaptation and natural selection (Ariani *et al.*, 2017; Rendón-Anaya *et al.*, 2017*b*; Cortés and Blair, 2018).

In this study, we hypothesized that drought is a factor that drives local adaptation in wild common bean. We observed that traits related to drought adaptation were associated phenotypically and genotypically with drought-related environmental factors affecting the populations in their location of origin. Conversely, domestication may have decreased tolerance to drought in common bean. Further corroboration of these findings in field environments is needed.

## MATERIALS AND METHODS

### Plant materials

**Fig. 2.**
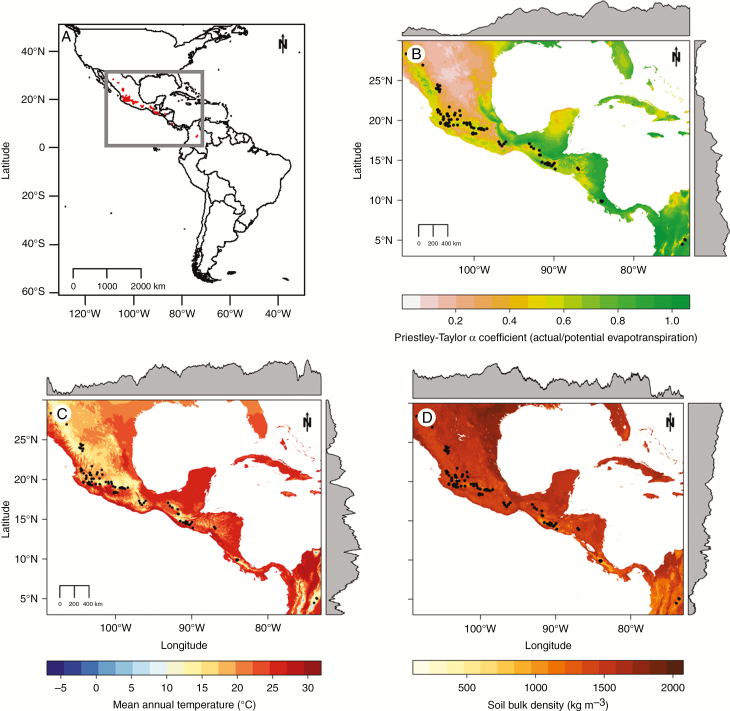
Location of origin of wild *Phaseolus vulgaris* accessions used in this study. (A) Mesoamerican wild bean accessions. (B) Close-up of Mesoamerica showing the Priestley–Taylor α coefficient. (C) Mean annual temperature. (D) Soil bulk density. The marginal plots in (B), (C) and (D) represent the longitudinal and latitudinal average and were plotted with the package raster (Hijmans and van Etten, 2016).

From the Mesoamerican gene pool of *Phaseolus vulgaris*, 112 wild accessions were selected to maximize geographical representation (Fig. 2). The seeds were obtained from the Genetic Resources Unit at the Centro Internacional de Agricultura Tropical (CIAT, Cali, Colombia), the National Genetic Resources Program (NGRP) of the USDA at the Western Regional Plant Introduction Station in Pullman, WA, USA, and from Jorge Acosta of the Instituto Nacional de Investigaciones Forestales, Agrícolas y Pecuarias (INIFAP, Mexico). Eleven reference domesticated Mesoamerican genotypes were included for comparison. SEA 5 (Singh *et al.*, 2001) and SER 118 were developed by CIAT, Flor de Mayo Eugenia (Acosta Gallegos *et al.*, 2010) and Pinto San Rafael (Acosta Gallegos *et al.*, 2016) by INIFAP, UCD 9634 by UC Davis (S. Temple, unpubl. res.), Matterhorn (Kelly *et al.*, 1999) and L88-63 (Frahm *et al.*, 2004) at Michigan State University, and Victor at Washington State University (Burke *et al.*, 1995). BAT 477, also developed at CIAT (Voysest 1983), was included as a drought-adapted check with a large root system (Sponchiado *et al.*, 1989; White and Castillo, 1992; Terán and Singh, 2002). IAC-Carioca 80SH from Brazil was included as a drought-sensitive control (Recchia *et al.*, 2013).

### Experimental setup

**Fig. 3.**
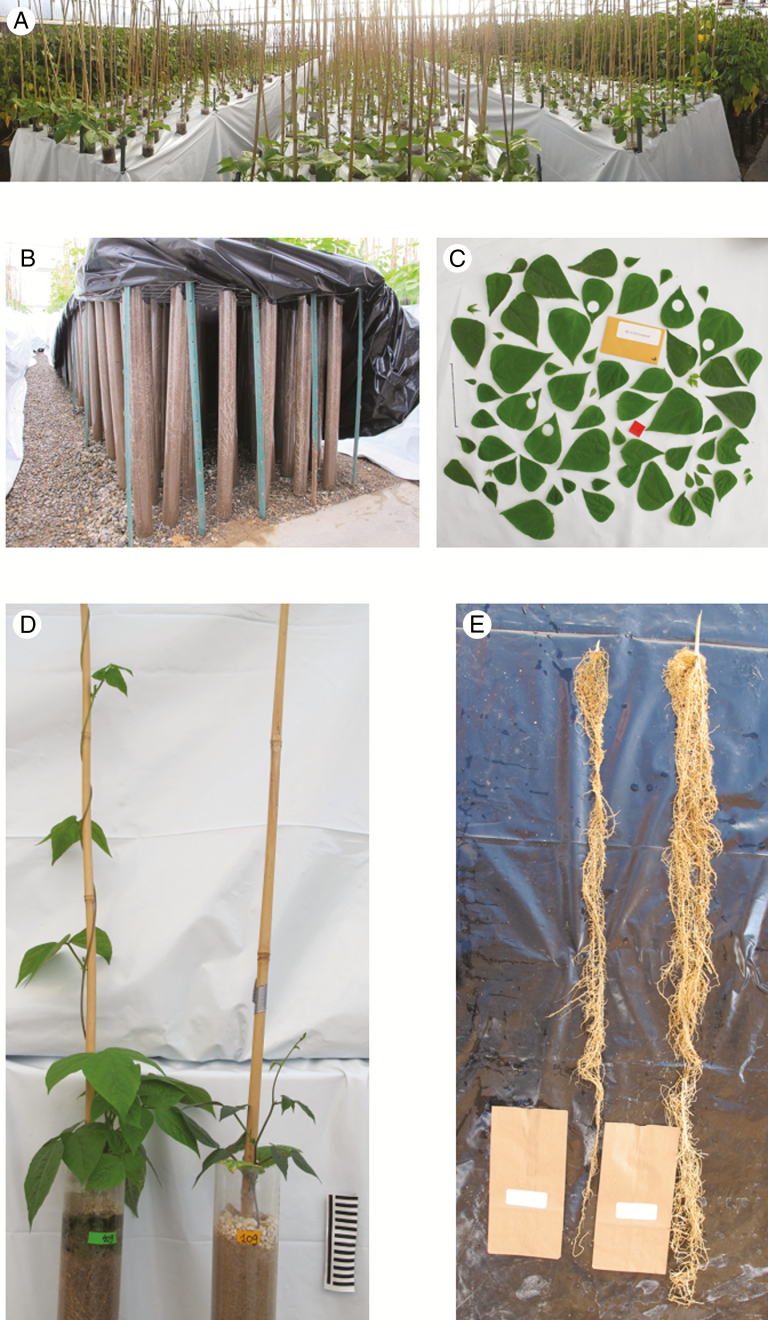
Wild bean growth experiment. (A) General overview of the experiment. (B) Root depth was observed through the experiment with clear plastic tubes. (C) Leaf sample collection with a known area sample (in red) for leaf area estimation. (D) Above-ground growth of the same genotype under full irrigation (left) and drought (right). (E) Root growth of the same genotype under drought stress (left) and full irrigation (right).

To observe root growth during the experiment, a screening method (Polanía *et al.*, 2009) was used with some modifications in a climate-controlled greenhouse (19–30 °C diurnal range, ~1000 µmol photons m^−2^ s^−1^ PAR at midday) in the Orchard Park Greenhouse facilities at the University of California-Davis (Fig. 3A). We used transparent tubes of 7.6 cm diameter and 1.2 m length made from polyethylene terephthalate (Fig. 3B; www.cleartecpackaging.com). The tubes were filled with a 2:1 sand:topsoil mix (bulk density of 1.70 g cm^−3^) up to 1 m high, with a similar amount of soil volume and compaction. A 1-cm layer of perlite was placed on the surface to reduce water loss through evaporation (Fig. 3B). The tubes were arranged in three blocks, each consisting of a wire grid structure 9.6 m long by 1.2 m wide, for a plant density of 24.3 plants m^−2^. The structure was covered in plastic (white exterior, black interior; Fig. 3A, B) to avoid root exposure to light and overheating. A 1.2-m long bamboo culm was placed at the centre of each tube for plant support.

The experimental design was a randomized complete block design, with two treatments and three replicates per treatment, and one plant per block–treatment combination. Blocks were planted on consecutive days. Two or three mechanically scarified seeds per genotype were planted in the tubes, one or two being later discarded to leave one plant after germination. The treatments consisted of full irrigation and irrigation withdrawal. For irrigation, the tubes were watered to field capacity at the time of planting and initial weight was recorded. Further irrigation to field capacity was given to both treatments until the expanded first trifoliate leaf stage (between V2 and V3), ~16 d after planting. The irrigation was continued in the irrigated treatment until the end of the experiment, while the drought treatment received no further irrigation. Plants were submitted to water stress for ~18 d. The drought treatment was applied to the vegetative stage only, immediately before reproductive initiation. This was performed to account for the non-synchronism in flowering initiation, especially among wild genotypes. Moreover, this vegetative stress represents a common intermittent water deficit in tropical and subtropical regions of Mexico and central and northern South America.

### Plant traits

After seeding, all tubes were monitored on a daily basis for time for emergence (the shoot was visible over the perlite layer; stage V1). The number of days to reach V3 stage was also recorded when the first trifoliate leaf was completely expanded. The number of days to reach each developmental stage was counted based on the day that the seed was placed in each block. Visual root depth and plant height were measured at an interval of 3–5 d (following the sequence used to plant each block). The relative amount of chlorophyll was measured 28 d after planting using a SPAD-502Plus Chlorophyll Meter (Konica-Minolta). At the end of the experiment, the above-ground biomass was collected, separating the leaves and stems. Leaf area was determined with Easy Leaf Area (Easlon and Bloom, 2014). Specific leaf area was calculated using six leaf punches with a known area (2.83 cm^2^) of the most recently expanded mature leaves (Fig. 3C), dried in a 50 °C oven for a week (Cornelissen *et al.*, 2003). At the end of the experiment, the roots were washed (Fig. 3E). Nodules and root whorl number were then counted and the roots were measured for their final length and dried.

The environmental variables [Priestley–Taylor α coefficient (PTAC), annual temperature and soil bulk density] were extracted from the geographical coordinates of the origin of each population using the package raster (Hijmans and van Etten, 2016). The PTAC is the ratio of actual to potential evapotranspiration, and integrates soil water availability assuming similar soil characteristics (Priestley and Taylor, 1972). The PTAC was obtained from the CGIAR-CSI database (Trabucco and Zomer 2010) (www.cgiar-csi.org), annual mean temperature from WorldClim (Hijmans *et al.*, 2005) (www.worldclim.org) and soil bulk density from SoilGrids (Hengl *et al.*, 2014) (www. soilgrids1km.isric.org). All variables were at 1-km resolution.

### Statistical analyses

The data were analysed using linear mixed models in R (R Development Core Team, 2017). Genotype, treatment (irrigated, drought) and their interaction were fixed effects. Blocks and their interaction with genotype and drought treatment were random effects. Statistical analyses were performed with the lme4 package (Bates *et al.*, 2015) and lmerTest to determine the significance of effects and to calculate least-squares means, using type-III hypothesis testing with Satterthwaite approximation for degrees of freedom. We determined *R*^2^ with the piecewiseSEM package (Nakagawa and Schielzeth, 2013). Marginal *R*^2^ is the variance explained by the fixed factors, while conditional *R*^2^ includes fixed and random factors. The coefficient of variation was calculated with sjstats (Lüdecke, 2016). Broad-sense heritability was estimated with REML (Holland *et al.*, 2003) as h2 =σG2/(σG2+σGT2+σE2), where σG2 is the genetic variance, σGT2 is the variance of the genotype × treatment interaction and σE2 is the variance of the experimental error. The correlation between traits was calculated only among wild samples and plotted with the corrplot package (Wei and Simko, 2016).

#### Environmental associations.

A Bayesian network analysis was performed with bnlearn (Scutari, 2012) to jointly analyse the phenotypic and environmental variables and the treatment effect. All continuous variables were jointly discretized to preserve the dependence structure while bypassing normality assumptions (Nagarajan *et al.*, 2013; Scutari and Denis, 2014). The continuous variables were discretized as multinomials with three levels using the hartemik method, and the treatment was left as a binomial. Structure learning was performed with a score base structure using the TABU greedy search. Average bootstrapping (10 000 iterations) with the Bayesian Dirichlet equivalent (bde) was used to obtain a consensus network. The nodes from phenotype towards environmental variables and treatment were blacklisted to improve stability. The trait ‘root whorl number’ was excluded due to the small phenotypic range, which did not allow proper discretization of the rest of the traits.

To evaluate the variation between genetic groups, the phenotypic and ecological data were analysed using linear mixed models in R (R Development Core Team, 2017). Genetic group (*n* = 3), treatment (irrigated, drought) and their interaction were considered fixed effects. The accessions were considered to belong to the group with the highest ancestry coefficient among the three groups. Statistical analyses were performed in the lme4 package (Bates *et al.*, 2015). The package lmerTest (Kuznetsova *et al.*, 2015) was used to estimate the effect significance using type-III hypothesis testing with Satterthwaite approximation for degrees of freedom and least-squares mean calculation.

#### Genotyping.

DNA was extracted from a random individual of each accession using a modified ammonium acetate-based protocol (Pallotta *et al.*, 2003). The 112 selected accessions were genotyped with 5398 single-nucleotide polymorphism (SNP) markers from the BARCBean6K_3 BeadChip SNP chip platform (Song *et al.*, 2015) at the USDA-ARS Soybean Genomics Improvement Laboratory, Beltsville, MD, USA. After filtering in GenomeStudio Module v1.8.4 (Illumina, San Diego, CA, USA), the SNP calling was performed automatically and with subsequent manual adjustments; after filtering for quality control and a 0.15 Gencall score cutoff, 5186 SNPs remained. The same accessions were also subjected to genotyping by sequencing (GBS) based on the *Cvi*AII enzyme (Ariani *et al.*, 2016). The reads were aligned to the G19833 reference genome version 2.1 [https://phytozome.jgi.doe.gov/pz/portal.html#!info?alias=Org_Pvulgaris (S. Jackson, P. McClean, J. Schmutz, unpubl. res.)] with BWA (Li and Durbin, 2009) and filtered with SAMtools (Li *et al.*, 2009) for minimum mapping quality of 10 and mean and maximum read depths of 5 and 1000, respectively. The joint dataset of the GBS and SNP chips consisted of 11 447 SNP markers.

#### Population structure and principal components analysis.

For population structure and principal components analyses, the SNP markers were pruned for linkage disequilibrium using PLINK (Purcell *et al.*, 2007) with a sliding window of ten SNPs, moving every other SNP and with a 0.5 *r*^2^ threshold, resulting in 2987 SNP markers. Population structure was analysed in TESS3, which includes spatial proximity information and least-squares optimization to improve accuracy and speed (Caye *et al.*, 2016). The number of ancestral populations was chosen from a sample of 1–20 using cross-validation. Principal components analysis was performed with the adegenet package (Jombart and Ahmed, 2011).

#### Genome-wide association analysis.

The 11 447 SNP markers were filtered for minor allele frequency of 10 % and maximum heterozygosity of 15 %, resulting in a total of 6755 markers. The relatively high stringency of the filtering for heterozygosity was chosen because common bean is a mostly self-fertilized species with relatively low rates of outcrossing (Triana *et al.*, 1993; Beebe *et al*., 1997b). Furthermore, under an additive model heterozygous markers do not have an effect, decreasing the statistical power of the marker, regardless of whether the heterozygosity is true or a genotyping error (Sham and Purcell, 2014). The missing data in the marker dataset were imputed with LinkImpute (Money *et al.*, 2015).

The genome-wide association analyses were performed for all the phenotypic traits evaluated and the three environmental variables. Box–Cox transformation (Box and Cox, 1964) was performed using the car package in R (Fox and Weisberg, 2010) to achieve normality of residuals. One random marker per chromosome was chosen in the model to evaluate the normality of residuals and to extract the optimal parameter for the transformation. Genome-wide association analysis was performed using the iterating Fixed And Random Model Circulating Probability Unification (FarmCPU) method (Liu *et al.*, 2016). Population structure and kinship were employed to account for the confounding between marker testing and relatedness estimation. The first three principal components were used as covariates to account for population structure. The principal components were extracted from the linkage disequilibrium-pruned dataset used for the population analyses. This procedure assists with possible over-representations of genomic regions and thus reduces the genome-wide ancestry representation signal (Price *et al.*, 2006). A nominal 5 % significance threshold with Bonferroni correction was used.

As other biotic and abiotic factors besides those studied here could also be driving local adaptation, a genome scan was performed to detect genomic regions under natural selection. The package pcadapt (Luu *et al*., 2017) was employed for detection of outlier markers in relation to population structure. Following the package’s recommendation, the first eight principal components were used to describe population structure under a range-expansion demographic model. Minor allele frequency was set at 10 %, as well as the Mahalanobis method for computing the *P*-values, and a false discovery rate of 0.1 %.

## RESULTS

### Experimental system for screening root and shoot growth under two watering regimes

Experiments were performed with wild and domesticated genotypes from the Mesoamerican gene pool only. Accessions of the Andean gene pool were not sampled due to the significant population structure within common bean (Blair *et al.*, 2009; Kwak and Gepts, 2009), which can increase the rate of spurious associations in genome-wide analyses (Kang *et al.*, 2008; Weigel *et al.*, 2015). Moreover, it has been suggested previously that, for common bean, genome-wide analyses should be done separately for each gene pool (Kwak and Gepts, 2009; Mamidi *et al*., 2011, 2013). Furthermore, the geographical and climatic ranges of Andean genetic diversity are smaller than those of the Mesoamerican pool (Schmutz *et al.*, 2014; Ariani *et al.*, 2017). Therefore, we selected 112 wild accessions of the Mesoamerican gene pool of *P. vulgaris*, representing the natural geographical distribution range of this gene pool (Fig. 2). Additionally, 11 domesticated genotypes, mostly elite cultivars known for their drought tolerance, high yield and other traits of interest, were included.

The wild and domesticated genotypes were phenotyped according to an experimental system (Polania *et al.*, 2009) performed in a greenhouse. Transparent tubes 1 m high were filled with a 2:1 sand:topsoil mix and arranged in three blocks (i.e. three replicates per genotype) in a wire-grid structure covered in plastic (white/black) to avoid root exposure to light and overheating during plant growth (Fig. 3A, B). The controlled environment of the greenhouse (range: night, 19 °C; day, 30 °C) and the completely randomized design were key factors for detecting significant (*P* < 0.05) differences among the treatments (control and drought) and genotypes (between wild and domesticated types and among wild forms).

### Above- and below-ground growth performance differences in wild and domesticated beans

**Fig. 4.**
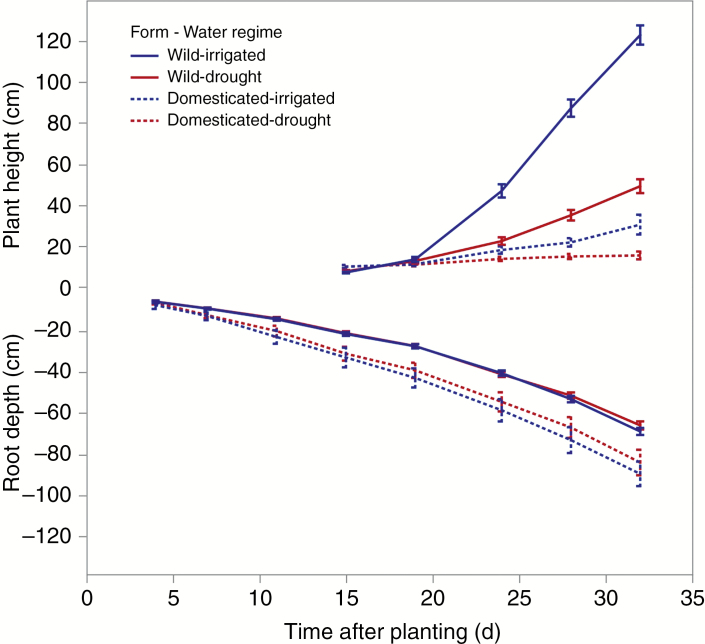
Plant height and visual root depth development. Plant height was measured starting 15 d after planting. The bars show the 95 % confidence interval.

Biomass, plant development and phenological traits (Table 1) were measured from planting to the V3 stage (first trifoliate), or over some 20–25 d, to assess the growth and development differences between wild and domesticated forms and the effect of drought on this development (Table 1). Domesticated beans were effective in developing deep roots in comparison with wild beans; in contrast, wild beans grew considerably taller than domesticated beans (Fig. 4). Under irrigated conditions, domesticated forms grew faster than wild types, achieving 75 % larger total biomass. Increased biomass accumulation in domesticated types was observed for all compartments, including leaves and roots. Domesticated types also achieved greater root depth and proportion of total biomass allocated to roots; however, they achieved lower root depth per unit of biomass (i.e. root exploration efficiency), indicating that the root system of domesticated forms was more spreading than that of wild types. Domesticated types, compared with wild forms, had a lower plant height and specific leaf area, and similar SPAD greenness value; they tended to have a larger leaf area and a higher root whorl number and nodule number (Table 1). Domesticated types were quicker to emerge after planting, but otherwise reached the V3 stage at a similar time compared with wild types.

**Table 1.** Analysis of variance and means for wild (W) and domesticated (D) forms under irrigated and drought conditions

Trait								Means				Heritability	*D*_d_ /*D*_i_	*W*_d_ /*W*_i_	(*D*_d_ /*D*_i_)/ (*W*_d_ /*W*_i_)
	Unit	*F* values				*R* ^2a^		Irrigated		Drought					
		Form^b^	Treatment	Form × treatment	CV (%)	Marginal	Conditional	D	W	D	W				
Biomass-related traits															
Stem biomass	g	26.2***	89**	30.2***	36.3	0.60	0.78	1.7	1.2	0.4	0.3	0.17	0.24	0.25	0.96
Leaf biomass	g	25.3**	114.1**	62.3***	29.5	0.70	0.84	3.2	2.1	0.6	0.5	0.12	0.19	0.24	0.79
Shoot biomass	g	26.9**	104.3**	51.9***	30.5	0.68	0.83	4.9	3.3	1.0	0.7	0.1	0.20	0.21	0.95
Root biomass	g	154.6***	49.2**	45.6***	42.8	0.35	0.58	2.9	1.2	1.5	0.8	0.08	0.52	0.67	0.78
Total biomass	g	82.2***	174.5**	63.4***	30.2	0.59	0.79	7.7	4.4	2.5	1.5	0.17	0.32	0.34	0.94
Root depth	cm	27.9***	2.4	0.4	21.0	0.40	0.41	89.7	69.0	84.3	66.0	0.13	0.94	0.96	0.98
Proportion of roots to total biomass		61.5***	110***	0.8	17.4	0.70	0.76	0.4	0.3	0.6	0.5	0.06	1.50	1.67	0.90
Root depth length per root biomass	cm g^−1^	36.9***	46.8***	0	37.1	0.16	0.51	34.0	73.2	68.1	107.9	0.05	2.00	1.47	1.36
Plant growth and development traits															
Specific leaf area	cm^2^ g^-1^	48.9***	75.7***	9.2*	16.0	0.42	0.49	362.1	463.2	306.7	348.7	0.08	0.85	0.75	1.13
Root whorl number		186.4***	1	0.2	30.6	0.36	0.47	2.6	1.3	2.6	1.2	0.14	1.00	0.92	1.09
SPAD		55.1***	183***	11.1*	9.2	0.72	0.76	37.7	31.0	46.5	43.3	0.07	1.23	1.40	0.88
Plant height	cm^2^	40.5**	123.6***	88.8***	25.4	0.62	0.80	33.7	124.3	17.1	49.7	0.36	0.51	0.40	1.28
Leaf area	cm^2^	4.1	74**	10**	37.2	0.65	0.80	1173.2	963.9	173.6	166.4	0.07	0.15	0.17	0.88
Nodule number		15.7***	32.2*	27.1***	77.7	0.43	0.63	100.2	60.5	5.3	5.6	0.04	5.28	9.26	0.57
Phenology															
Time from planting to emergence	d	5.4*	0.1	0.1	15.5	0.30	0.60	6.0	6.6	5.9	6.6	0.32	0.98	1.00	0.98
Time from planting to V3	d	10.2	66.9***	53.8***	9.3	0.37	0.65	20.4	19.6	25.7	20.7	0.19	1.26	1.01	1.25
Time from emergence to V3	d	24.4**	120.8***	72.1***	12.3	0.46	0.56	14.4	13.0	19.8	14.1	0.07	1.38	1.08	1.28

CV, coefficient of variation.

*P < 0.05; **P < 0.05; ***P < 0.01.

^a^Marginal *R*^2^ is the variance explained by the fixed factors; conditional *R*^2^ includes fixed and random factors.

^b^Wild versus domesticated.

The drought regime resulted in a stress large enough to reduce biomass and plant height growth (averaged over wild and domesticated types; Figs 3D, E and 4) by two-thirds, suggesting that the stress struck an appropriate middle ground for differentiation purposes. Under this regime, the response of domesticated types relative to wild forms was similar to results under well-watered conditions, with several exceptions. Exceptions include an increase in the time from emergence to the V3 stage in domesticated relative to wild types and a sharp reduction in the number of nodules, such that both wild and domesticated types showed the same low level of nodules under drought. Particularly noteworthy, however, was that both the domesticated and the wild type showed an increase in the proportion of root to total biomass. When comparing only wild beans, there was significant phenotypic variation in all traits except specific leaf area (Table 1). The biomass traits were affected by the drought treatment and their interaction with genotype. Root depth was not affected by drought, but the proportion of roots to total biomass, plant height, specific leaf area and nodule number were highly affected by drought. Genotype × treatment interaction was significant (*P* < 0.05) for biomass-related traits, plant height and nodule number.

To compare the effect of the drought stress imposed on domesticated versus wild types, we calculated the ratio (*D*_d_/*D*_i_)/(*W*_d_/*W*_i_), where *D*_d_ and *D*_i_ are the average values of a measured trait under drought and irrigated conditions, respectively, in the domesticated group, and *W*_d_ and *W*_i_ are the corresponding values for the wild group (Table 1). Values close to 1 suggest similar effects of drought stress in wild and domesticated beans; in contrast, the more the value of this ratio differs from 1 (either below or above this value), the more the response to drought differs between wild and domesticated types. Our results show that three traits showed a markedly larger response to drought stress in the domesticated group compared with the wild group: root depth length per biomass unit, plant height and time from emergence to V3 (Table 1). The domesticated types increased the depth of roots relatively more per unit of root biomass than wild types. Yet even under this drought stress the root depth per biomass unit was 60 % greater in the wild than in the domesticated types. Plant height was reduced more in wild types than in domesticated forms because of the drought stress imposed. In contrast, domesticated types increased the duration of the emergence-to-V3 stage by 5 d, whereas in wild types it increased by only 1 d.

Two traits decreased less in wild types because of the drought regime imposed: leaf and root biomass. In domesticated types, leaf and root biomass were reduced by 80 and 50 %, respectively. On average, wild types may offer tolerance to drought primarily because of an ability to continue early growth and development in spite of drought, as shown by minimal delay in reaching the V3 stage and smaller reduction in shoot and root biomass accumulation. This analysis does not address other mechanisms of tolerance that may operate at later growth and development stages.

**Fig. 5.**
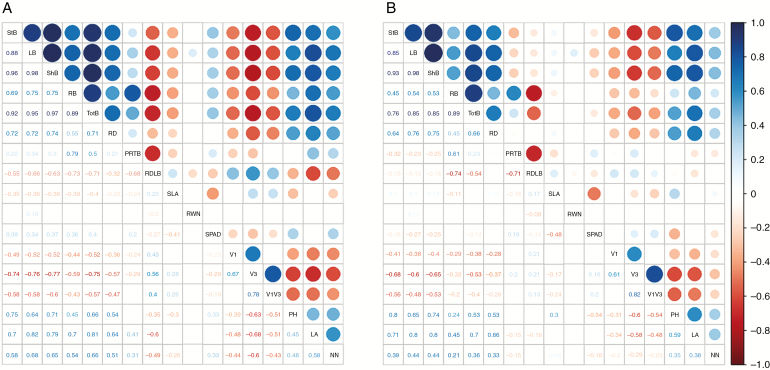
Phenotypic correlation among traits. (A) Irrigated. (B) Drought. Only correlations significant at *P* < 0.05 are shown. BLD, soil bulk density; LA, leaf area; LB, leaf biomass; NN, nodule number; PH, plant height; PRTB, proportion of roots to total biomass; PTAC, Priestley–Taylor α coefficient; RB, root biomass; RD, root depth; ShB, shoot biomass; SLA, specific leaf area; StB, stem biomass; SPAD, chlorophyll content; Temp, temperature; RDLB, root depth length per biomass; TotB, total biomass; V1, time to emergence; V3, time from planting to V3; V1V3, time from emergence to V3. The colours represent that the values of the correlation coefficient according to the heat map to the right of the graph. The size of of the circles is proportional to the absolute value of the correlation coefficients.

After computing heritability estimates for the traits, relatively low values were encountered; the range was from 0.04 (nodule number) to 0.36 (plant height) (Table 1). Among the biomass traits, total biomass showed the highest heritability. Correlation analyses among all traits were also computed. High correlation was detected among some traits, and their patterns were similar in the two watering treatments (Fig. 5). Total biomass was positively correlated with root depth, proportion of roots to total biomass, SPAD, leaf area, plant height and nodule number, but negatively with specific leaf area. Root depth was positively correlated with plant height, leaf area and nodule number.

### Deeper-rooted and more productive wild beans are associated with the driest environments of origin

**Fig. 6.**
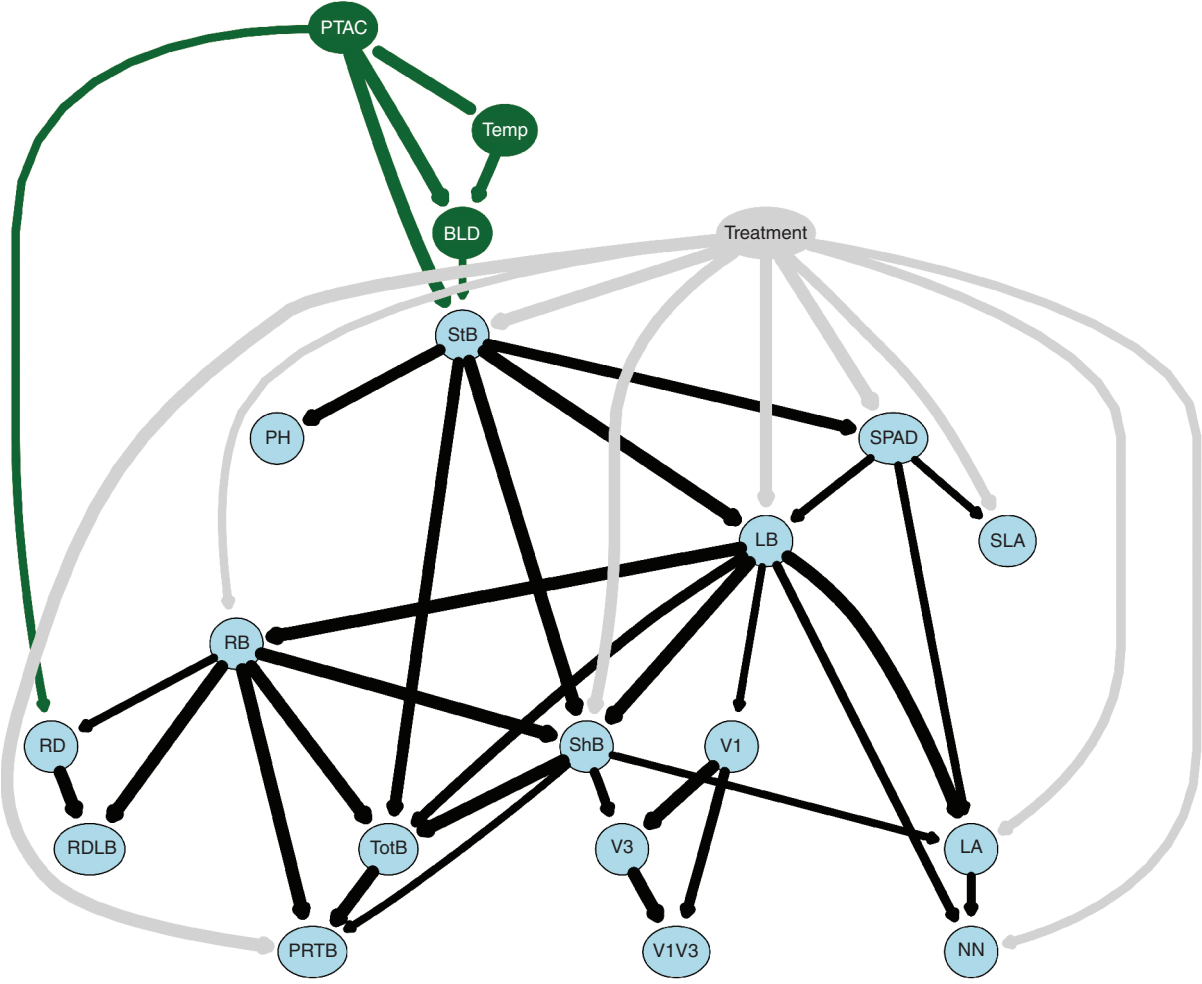
Average network analysis from cross‐validation by bootstrapping (10 000 iterations). The plot only includes nodes above the significance threshold and line thickness represents the strength of the relationship. Phenotypic traits are coloured in blue, environmental variables in green and the treatment node (well watered versus drought-stressed) in grey. Abbreviations are defined in the legend of Fig. 5.

The drought responses observed were hypothesized to relate to the local environment in which the wild bean accessions originated. The environmental variables included the PTAC, annual temperature and soil bulk density. A Bayesian network approach was employed to jointly analyse the phenotypic and environmental variables and the treatment effect and to allow more exploratory inferences than were obtainable by path-specified diagrams. The resulting network comprised 20 nodes and 44 arcs (Fig. 6). The treatment effect influenced shoot and root biomass traits, SPAD and nodulation capacity, but, surprisingly, not root depth or phenology. The environmental variables, particularly PTAC, influenced directly just two plant responses (stem biomass and root depth), which in turn influenced other plant characteristics.

**Fig. 7.**
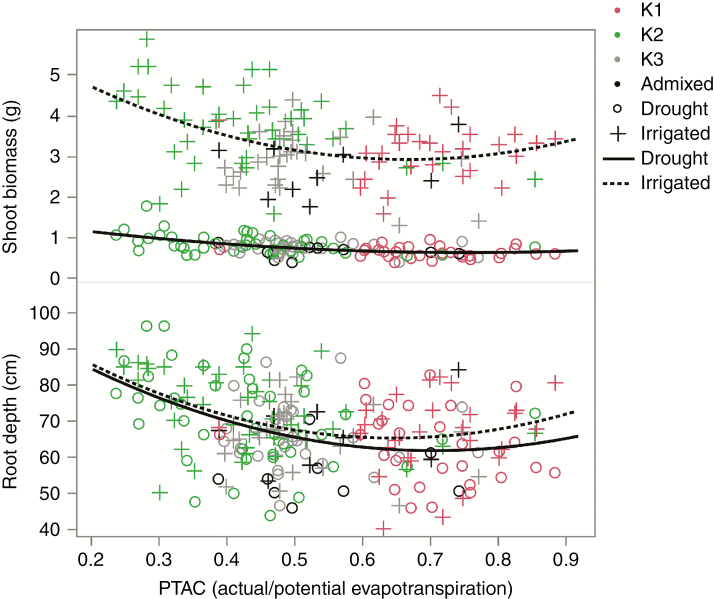
Linear and quadratic regression analyses of shoot biomass and root depth as explained by aridity (PTAC) in irrigated (dotted lines) and drought (solid lines) treatment. The colours of the points represent the population groups identified by structure analysis.

Regression analysis between the two variables directly associated with PTAC, stem biomass and root depth, showed linear and quadratic negative associations, with the deeper-rooting and highly productive accessions originating from the driest environments (Fig. 7). Furthermore, the variation explained by PTAC was higher when the phenotypes were measured under drought than under irrigation for both shoot biomass (*R*^2^ of 0.24 versus 0.1) and root depth (*R*^2^ of 0.16 versus 0.14). Soil bulk density directly influenced stem biomass. Temperature was directly related to both environmental variables, but not directly to any phenotypic variable.

### Wild beans from the driest environments are also structured at the genomic level

**Fig. 8.**
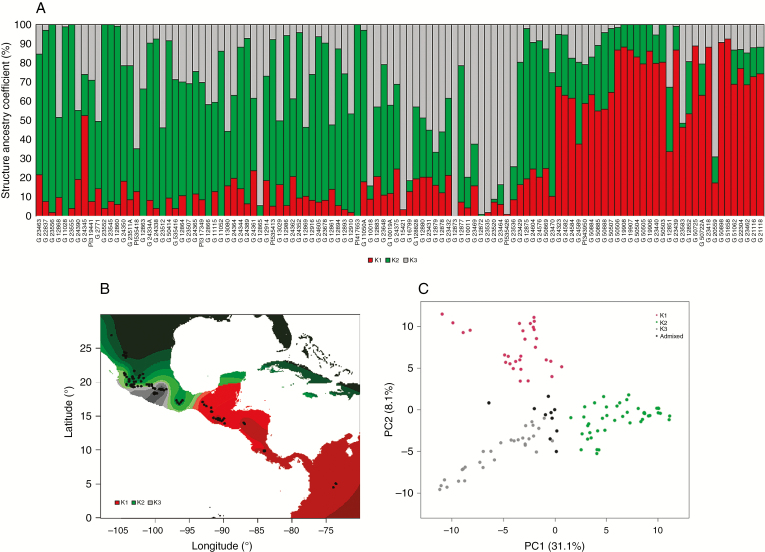
Structure analysis of Mesoamerican wild bean SNP diversity. (A) Barplot of structure ancestry coefficients, sorted by longitude from north to south. (B) Geographical map of ancestry coefficients. (C) Principal components analysis, coloured by structure groups. Accessions with a cluster coefficient cut-off <70 % are coloured as admixed. PC1, PC2, principal components 1 and 2. The percentages in the axis labels represent the eigenvalues of the principal components.

The next level of our analysis consisted of genotyping SNP markers for all the wild accessions and determining their population structure. Using the BARCBean6K_3 BeadChip SNP chip platform (Song *et al.*, 2015) and GBS based on the *Cvi*AII enzyme (Ariani *et al.*, 2016), 11 447 SNP markers were available after filtering for quality. After pruning the markers for linkage disequilibrium, 2987 SNP markers were included in the analysis. The population structure analysis suggested the existence of three Mesoamerican wild populations (K1, K2 and K3) (Fig. 8). Population K1 was located from Mexico, south of the Isthmus of Tehuantepec, throughout Central America to Colombia; population K2 was distributed in central–northern Mexico (states of Jalisco, Guanajuato, Nayarit, Zacatecas, Durango and Chihuahua) and the southern state of Oaxaca. Population K3 was distributed in central–west Mexico, mainly in the state of Guerrero (Fig. 8). A principal components analysis showed two groups along the first component (31 % of the variation), the first one comprising K1 and K3; K2 constituted the second group. The second principal component (8 % of the variation) separated K1 and K3. The number of groups is similar to those identified in previous studies, i.e. from two to four (Kwak and Gepts, 2009; Bitocchi *et al.*, 2012; Blair *et al.*, 2012).

When comparing the genetic groups, the group effect was significant for all traits, except for root depth length per biomass, root whorl number, SPAD and time to emergence (Table 2). For all biomass traits (except root depth), all plant growth and development traits, and all phenology traits (except time to emergence), the effects of treatment and its interaction were significant. For the three environmental variables, there were significant differences among groups. In general, the group K2 of northern Mexico and Oaxaca showed deeper rooting and higher biomass, leaf area (but lower specific leaf area), plant height and nodule number. In addition, this group originated in drier areas (low PTAC), with lower temperatures and higher soil bulk density.

**Table 2.** Analysis of variance and means of the effects of genetic groups (K1–K3) and watering treatments

Trait	Unit	*F* value			*R* ^2^	Mean^a^					
		Group	Treatment	Group × treatment		K1		K2		K3	
Stem biomass	g	13.9***	574***	5.8**	0.76	1.2	B	1.4	A	1.2	B
Leaf biomass	g	7.7***	1016***	4.6*	0.84	0.6	B	0.8	A	0.6	B
Shoot biomass	g	10.5***	875.8***	5.5**	0.82	1.9	B	2.2	A	1.8	B
Root biomass	g	8.7***	54.2***	5.3**	0.30	1	A	1.1	A	0.8	B
Total biomass	g	10.2***	520.5***	6.5**	0.74	2.8	B	3.3	A	2.6	B
Root depth	cm	7.3***	2.3	0.6	0.08	64.7	B	70.7	A	65.5	B
Proportion of roots to total biomass		9***	1202.7***	5.1**	0.85	0.4	A	0.4	B	0.4	B
Root depth length per biomass	cm g^−1^	2.7	73.5***	3.3*	0.31	85.7	A	89.5	A	96.7	A
Specific leaf area	cm^2^ g^−1^	3.3*	453.4***	5.4**	0.68	409	AB	399	B	416	A
Root whorl number		2.2	6.2*	0.5	0.06	1.3	A	1.3	A	1.2	A
SPAD		0.6	1370.7***	6.1**	0.87	37.2	A	37.3	A	36.9	A
Time to emergence	d	1.1	0.1	0.3	0.01	6.7	A	6.5	A	6.7	A
Time from planting to V3	d	9***	30.4***	2	0.20	20.7	A	19.7	B	20.5	A
Time from emergence to V3	d	10.1***	52.2***	3.3*	0.27	14	A	13.2	B	13.8	A
Plant height	cm^2^	29.2***	658.8***	0.9	0.78	72.3	C	97.9	A	83.5	B
Leaf area	cm^2^	3.5*	969.5***	2.2	0.83	558	AB	596	A	520	B
Nodule number		6.4**	334.4***	3.9*	0.65	28.3	B	38.4	A	28.6	B
Priestley–Taylor α coefficient		56.2***			0.53	0.7	A	0.4	C	0.5	B
Annual temperature	°C	9***			0.15	19.1	B	18.3	B	20.8	A
Soil bulk density	kg m^−3^	35.2***			0.42	1011	B	1238	A	1212	A

### Genomic regions associated with drought-related traits and environmental variables are probably under selection in both wild and domesticated types

**Fig. 9.**
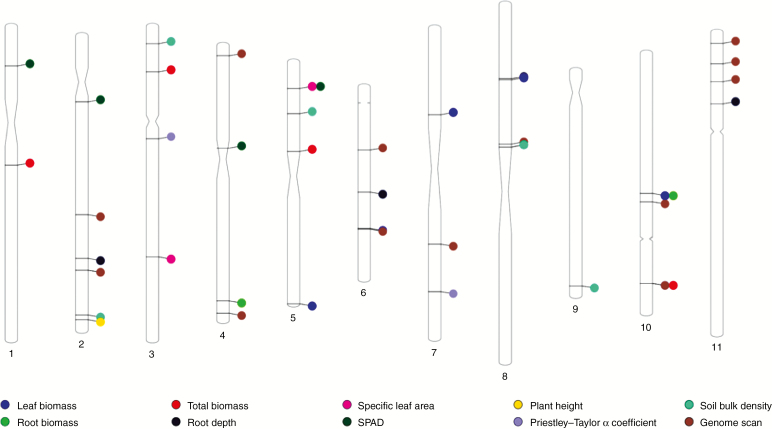
Chromosomal ideogram of common bean. Significant markers in the genome association analysis of phenotypic and ecological traits and the genome scan are shown.

Genome-wide association analyses were performed to uncover molecular markers associated with phenotypic traits and environmental variables among wild accessions. In total, 6755 SNP markers from BARCBean6K_3 BeadChip and the GBS were used in the genome-wide association study, using the Fixed and Random Model Circulating Probability Unification 260 (FarmCPU) method (Liu *et al.*, 2016). These analyses identified 23 SNPs that were associated with phenotypic traits and seven with the environmental variables (Supplementary Data Table S1). Among the phenotypic variables, leaf biomass, root biomass, total biomass, root depth, specific leaf area, SPAD and plant height were associated with six, four, four, four, two, four and one SNP markers, respectively. Among the environmental traits, two significant SNPs were found for PTAC and six for soil bulk density; surprisingly, none were found for annual temperature. Considering that other biotic and abiotic factors could also be driving local adaptation, a genome scan was performed to detect genomic regions under natural selection. Fifteen SNP markers were found under selection in the genome scan. The SNPs were found on all 11 chromosomes (Supplementary Data Table S1 and Fig. 9).

## DISCUSSION

The evaluation of wild beans through phenotyping methods and the association among phenotypes and ecological and genetic variation has been sparse because wild beans have a vigorous growth habit, long growth cycle and photoperiod sensitivity, which make a complete evaluation impractical. In addition, a representative collection in gene banks was until recently unavailable (Ariani *et al.*, 2017). We deployed a greenhouse screening method as a common garden experiment to evaluate and compare 112 accessions of wild common bean accessions belonging to the Mesoamerican gene pool with domesticated counterparts for key growth responses presumably related to drought adaptation. The association of growth variables with descriptors of the environment of origin would indicate local adaptation to drought. Furthermore, associations at the phenotypic, genotypic and genomic levels provide an assessment of the molecular basis of adaptation to drought in wild populations. To the best of our knowledge, this in-depth and extensive analysis of drought adaptation is the first of its kind in the wild relative of a crop.

### Wild and domesticated phenotypic evaluation

We justify conducting a greenhouse experiment, rather than a field experiment, to measure different phenotypic traits on the following basis. First, some traits are difficult to measure in the field, especially root traits. While the tube setup and the soil matrix are different from what one would find in any field, they nevertheless allowed us to measure total root biomass and root depth, the latter to >80 cm depth. Second, wild types are genetically more differentiated than domesticated types, including for adaptation (Papa and Gepts, 2003). There has been a broadening of the adaptation of domesticated beans compared with that of wild beans. It is therefore easier to conduct common-garden-type experiments for wild types in conditions like those of a greenhouse, where the range of temperatures and irrigation can be controlled.

One of the key findings was the effect that drought promoted deeper root growth. At the same time, canopy growth was suppressed but comparatively less change in root biomass was observed (Table 1, Figs 4 and 7). Based on other lines of evidence, this would reflect soil water deficit avoidance (Gilbert and Medina, 2016). When comparing wild types with their domesticated counterparts, domesticated forms were more productive in terms of biomass and rooting depth. Selection during domestication and crop development increased both root and shoot mass, root depth and the proportion of biomass invested in roots, as found in *Pisum sativum* (Weeden, 2007) and wheat (Waines and Ehdaie, 2007). However, in this research wild accessions were more efficient at reaching deeper soil strata relative to the amount of biomass invested in roots, suggesting that competition for soil water may be higher in wild than in domesticated forms (Table 1).

Domesticated beans had more root whorls than the wild types, as found previously (Miguel *et al.*, 2013). The higher number of root whorls is positively associated with phosphorus acquisition efficiency as it changes root architecture by increasing root biomass and promotes soil exploration in the superficial soil layers, where phosphorus is mostly distributed (Miguel *et al.*, 2013). This observation strengthens the hypothesis that phosphorus efficiency (acquisition and use) increased during domestication (Beebe *et al*., 1997a; Miguel *et al.*, 2013). Beans, along with maize and squash, were domesticated and integrated into the milpa mixed crop system (Zizumbo-Villarreal and Colunga-GarcíaMarín, 2010). Under these conditions, the root architectures of these three crops are spatially complementary and avoid interspecific competition for immobile nutrients (Postma and Lynch, 2012), but beans can explore the soil profile better than maize and squash (Zhang *et al.*, 2014).

After analysing the correlations among traits, similar results were obtained in the two treatments (well-watered and drought). Root depth was positively correlated with biomass production and plant height. The correlation and importance of deep roots for high productivity in biomass and grain yield under drought has been described for domesticated common beans under field and greenhouse conditions (Sponchiado *et al.*, 1989; Polanía *et al*., 2017). While having a large leaf area might be disadvantageous under drought conditions, canopy biomass (especially considering the number of nodes and leaves) is positively associated with grain yield (Tanaka and Fujita, 1979; Sponchiado *et al.*, 1989; Polanía *et al*., 2016, 2017). Although plants did not achieve reproductive maturity in this experiment, we assume that wild genotypes that produce more above-ground biomass, especially under drought conditions, would have higher seed number and grain yield, as a measure of fitness.

Even under drought stress, wild types had roots that reached 60 % deeper (per unit of root biomass) than those of domesticated types. Furthermore, wild accessions were superior under drought stress compared with their domesticated descendants by showing less delay in reaching the V3 stage and a smaller reduction in root and shoot biomass, suggesting a stronger capacity to achieve biomass growth even under stress (Table 1). As an example, wild genotypes G22837 from Chihuahua and G24576 from Oaxaca showed consistent deep rooting capacity and high biomass production across treatments. These genotypes could be candidates for inclusion in breeding programmes. Interestingly, although they originated relatively far from each other, one from the northernmost distribution of wild *P. vulgaris* in northern Mexico and the other from western Mexico, both belong to the same genetic group and originated in dry environments, with 536 mm (for G22837) and 581 mm (for G24576) of annual rainfall, respectively, while the across-accession average in this study was 1125 mm of annual rainfall.

### Association of genomic regions associated with phenotypes related to drought adaptation and ecological factors

Under our hypothesis of the existence of local adaptation in wild common beans, driven by drought and other abiotic factors, finding phenotypic traits associated with specific genomic locations should be expected. Markers in the genome reflecting environmental differentiation/stresses among populations should be co-located with markers controlling phenotypic responses presumably involved in adaptation to these stresses. In the genome-wide association analyses, we found 23 SNPs that were associated with phenotypic traits: leaf biomass, root biomass, total biomass, root depth, specific leaf area, SPAD and plant height. Two SNPs were associated with PTAC and six with soil bulk density, but none with temperature. In addition, 15 SNPs were found to be under selection after conducting a genome scan. We found overlapping regions between phenotypic traits, climatic variables and the genome scan analyses. This suggests either close linkage or pleiotropy (Denny *et al.*, 2016; Saltz *et al.*, 2017). For example, an SNP on chromosome Pv10 was shared between traits related to root and leaf biomass. This SNP is also close (1.4 Mb) to an SNP found in the genome scan. It is possible that within this genomic region there are one or more genes that control plant growth and also show a signature of selection among our samples.

A literature survey was carried out to compare and find previous quantitative trait locus (QTL) analyses that overlap with the regions of the present study. An SNP for SPAD on chromosome Pv02 is located 0.79 Mb from the closest marker for a QTL that controls yield, seed weight, pod wall ratio, biomass weight and normalized difference vegetation index (NDVI) in a domesticated × domesticated population (Trapp *et al.*, 2016). NDVI is also a measure of greenness, a proxy of chlorophyll content, similar to the SPAD measure (Adamsen *et al.*, 1999). A plant height SNP on chromosome Pv02 is 0.61 Mb away from a shoot biomass SNP in a genome-wide association study of race Mesoamerican germplasm (Hoyos-Villegas *et al.*, 2016). A leaf biomass SNP on chromosome Pv05 is 1.57 Mb from an SNP for seed yield (Trapp *et al.*, 2016) and 3.7 Mb from the closest marker of a QTL for seed yield in a wild × domesticated backcrossed recombinant inbred line population (Blair and Izquierdo, 2012). An SNP from the genome scan on chromosome Pv07 was located between two SNPs associated with seed yield QTLs, at a distance of 2.2 and 2.1 Mb, respectively, in a nested association mapping (NAM) of domesticated Mesoamerican beans (Hoyos-Villegas *et al.*, 2016). On chromosome Pv08, SNPs of leaf biomass and root depth were located 3.5 and 3.7 Mb away from the closest marker of a QTL for shoot biomass in a domesticated recombinant inbred population, in an experimental setting similar to our work (Asfaw and Blair, 2012). The SNP on chromosome Pv10 that was associated with both root and leaf biomass is 2.2 and 2.74 Mb from markers close to QTLs for seed yield and canopy height, respectively, in an NAM population (Hoyos-Villegas *et al.*, 2016). The overlap of these regions suggests that they are under selection in both wild and domesticated populations. These regions are of interest for fine mapping, subsequent cloning and introgression in breeding programmes, as are other regions found in the wild germplasm that have not been identified in domesticated panels.

The present research is the first genome-wide association analysis at the phenotypic level in this species using solely wild specimens. It is possible that, due to a genetic bottleneck during domestication, some of the beneficial wild alleles were not present in the populations where domestication took place (Gepts *et al.*, 1986; Sonnante *et al.*, 1994; Tanksley and McCouch 1997). In addition, it might also be possible that new beneficial or detrimental mutations arose during crop improvement after domestication (Moyers *et al.*, 2018). Previous efforts in detecting beneficial wild variation was performed using wild × domesticated recombinant inbred populations, in addition to domesticated and wild panels for genome-wide studies (Mickelbart *et al.*, 2015). Recently, 86 wild accessions were studied for genomic–environmental association; various genomic regions associated with bioclimatic variables were identified (Cortés and Blair, 2018). Nevertheless, the smaller sample size, the presence of a strong population structure due to the inclusion of both Andean and Mesoamerican gene pools as well as a sister species (Rendón-Anaya *et al.*, 2017*a*), and high spatial–genetic–environmental autocorrelation (Papa and Gepts, 2003; van Heerwaarden *et al.*, 2010; Lotterhos and Whitlock, 2015; Thurman and Barrett, 2016), and the reliance on a single drought parameter suggest the need for more careful choice of plant materials and more detailed phenotyping. Finally, it may be worthwhile screening domesticated bean germplasm for the same traits putatively involved in drought stress tolerance of wild beans. Domesticated beans harbouring such traits may provide an alternative genetic background to use in breeding programmes.

### Conclusions

Domesticated beans were more vigorous in general: they produced more biomass, above and below ground, and developed deeper roots. However, wild beans showed reduced phenological delay as well as smaller reductions in root and shoot biomass accumulation under drought stress, traits that could be useful to improve the domesticated gene pool. In this regard, the genetic groups from northern Mexico and Oaxaca are candidate germplasm sources for pre-breeding activities to improve drought adaptation in commercial cultivars, because of the greater aridity of the area of origin and their biomass response and deeper roots under drought. We found genomic regions that were associated with productivity and drought adaptation in the wild germplasm. Further research is needed to validate and dissect these genomic regions. Field experiments are also necessary to further analyse phenotypic–genotypic associations. Introgression of these regions into domesticated genetic backgrounds could be used to assess whether their effects are beneficial in a domesticated background. The specific molecular mechanisms underlying such interactions are yet to be uncovered.

## SUPPLEMENTARY DATA

Supplementary data are available online at https://academic.oup.com/aob and consist of Table S1: list of significant SNP markers related to phenotypic and environmental traits and the genome scan.

## Supplementary Material

mcy221_suppl_Supplementary_Material
