# Supplementary material for: Root and shoot variation in relation to potential intermittent drought adaptation of Mesoamerican wild common bean (Phaseolus vulgaris L.)
Source: Ann Bot. 2018 Dec 31;124(6):917–32. doi: 10.1093/aob/mcy221 (PMC6881220; doi:10.1093/aob/mcy221)
Supplement: mcy221_suppl_Supplementary_Material [file mcy221_suppl_supplementary_material.docx]

| Supplementary Table 1. List of significant SNP markers related to phenotypic, environmental traits and genome scan. | | | | |
| --- | --- | --- | --- | --- |
| **Trait** | **Marker** | **Chromosome** | **Position** | **P.value** |
| Leaf biomass | BARCBEAN6K_3-SNP1142 | 5 | 40271614 | 4.33E-08 |
| Leaf biomass | SNP_7468 | 10 | 23391592 | 4.77E-08 |
| Leaf biomass | BARCBEAN6K_3-SNP1343 | 10 | 38302716 | 3.29E-07 |
| Leaf biomass | BARCBEAN6K_3-SNP1349 | 8 | 12658595 | 4.50E-07 |
| Leaf biomass | SNP_5008 | 7 | 14449663 | 5.28E-06 |
| Leaf biomass | SNP_4539 | 6 | 23568560 | 7.03E-06 |
| Root biomass | BARCBEAN6K_3-SNP0669 | 4 | 42530005 | 2.65E-06 |
| Root biomass | SNP_7468 | 10 | 23391592 | 7.58E-06 |
| Total biomass | BARCBEAN6K_3-SNP1343 | 10 | 38302716 | 3.26E-08 |
| Total biomass | BARCBEAN6K_3-SNP3167 | 1 | 23155761 | 4.19E-07 |
| Total biomass | BARCBEAN6K_3-SNP4954 | 3 | 7616378 | 1.90E-06 |
| Total biomass | BARCBEAN6K_3-SNP3745 | 5 | 14894789 | 5.23E-06 |
| Root depth | BARCBEAN6K_3-SNP1351 | 8 | 12457029 | 5.15E-09 |
| Root depth | BARCBEAN6K_3-SNP3223 | 6 | 17528543 | 6.33E-07 |
| Root depth | SNP_8004 | 11 | 11902160 | 3.73E-06 |
| Root depth | SNP_1359 | 2 | 37085409 | 5.02E-06 |
| Specific Leaf Area | SNP_2235 | 3 | 38425720 | 2.19E-07 |
| Specific Leaf Area | BARCBEAN6K_3-SNP2241 | 5 | 4411502 | 3.17E-06 |
| SPAD | SNP_2851 | 4 | 17085311 | 3.99E-06 |
| SPAD | BARCBEAN6K_3-SNP2241 | 5 | 4411502 | 5.33E-06 |
| SPAD | SNP_995 | 2 | 11004739 | 8.19E-06 |
| SPAD | BARCBEAN6K_3-SNP2535 | 1 | 6589660 | 9.05E-06 |
| Plant height | SNP_1578 | 2 | 47301929 | 4.45E-07 |
| PTAC | SNP_1972 | 3 | 18749238 | 1.26E-07 |
| PTAC | SNP_5287 | 7 | 43926437 | 1.34E-06 |
| Soil bulk density | BARCBEAN6K_3-SNP4876 | 5 | 8593465 | 4.95E-11 |
| Soil bulk density | BARCBEAN6K_3-SNP2827 | 3 | 2894554 | 1.52E-08 |
| Soil bulk density | SNP_7016 | 9 | 35822398 | 2.43E-08 |
| Soil bulk density | SNP_1563 | 2 | 46511718 | 4.83E-07 |
| Soil bulk density | SNP_5867 | 8 | 23827450 | 6.70E-06 |
| Genome scan | SNP_1241 | 2 | 29796820 | 2.77E-06 |
| Genome scan | SNP_1398 | 2 | 39027393 | 7.37E-08 |
| Genome scan | SNP_1399 | 2 | 39027395 | 3.16E-07 |
| Genome scan | SNP_2477 | 4 | 1723447 | 3.09E-09 |
| Genome scan | SNP_3296 | 4 | 44619109 | 6.26E-07 |
| Genome scan | SNP_4114 | 6 | 10515936 | 1.76E-06 |
| Genome scan | SNP_4543 | 6 | 23724503 | 7.71E-11 |
| Genome scan | SNP_5172 | 7 | 36046974 | 0.0000027 |
| Genome scan | SNP_5862 | 8 | 23326725 | 1.73E-06 |
| Genome scan | SNP_5863 | 8 | 23827401 | 1.73E-06 |
| Genome scan | BARCBEAN6K_3-SNP3417 | 10 | 24745230 | 3.00E-14 |
| Genome scan | BARCBEAN6K_3-SNP1342 | 10 | 38310396 | 4.64E-08 |
| Genome scan | BARCBEAN6K_3-SNP0199 | 11 | 1820186 | 7.79E-08 |
| Genome scan | BARCBEAN6K_3-SNP2902 | 11 | 5237195 | 7.79E-08 |
| Genome scan | BARCBEAN6K_3-SNP3593 | 11 | 8211793 | 7.79E-08 |
